# Global School-based Student Health Survey reveals correlates of suicidal behaviors in Brunei Darussalam: a nationwide cross-sectional study

**DOI:** 10.5249/jivr.v12i3.1371

**Published:** 2020-08

**Authors:** Nasrin Shahedifar, Masood A. Shaikh, Frederick Oporia, Michael Lowery Wilson

**Affiliations:** ^*a*^ Road traffic Injury Research Center, Tabriz University of Medical Sciences, Tabriz, Iran; Injury Epidemiology and Prevention Research Group, Turku Brain Injury Center, Division of Clinical Neurosciences, Turku University Hospital and University of Turku, Turku, Finland.; ^*b*^ Injury Epidemiology and Prevention Research Group, Turku Brain Injury Center, Division of Clinical Neurosciences, Turku University Hospital and University of Turku, Turku, Finland.; ^*c*^ Department of Disease Control and Environmental Health, Makerere University School of Public Health, Kampala Uganda; Injury Epidemiology and Prevention Research Group, Turku Brain Injury Center, Division of Clinical Neurosciences, Turku University Hospital and University of Turku, Turku, Finland.; ^*d*^ Heidelberg Institute of Global Health (HIGH), University of Heidelberg, Heidelberg, Germany.

**Keywords:** Suicide, Adolescents, Global School Based Student, Health Survey, Brunei Darussalam

## Abstract

**Background:**

This study aimed to determine the prevalence of and correlates for suicidal behaviors among school attending adolescents in Brunei.

**Methods:**

Nationally representative cross-sectional data on (n=2599) adolescents derived from the Global School-based Health Survey in Brunei Darussalam were examined. Data on suicidal behaviors, psychosocial and demographic characteristics were analyzed using multiple logistic regression taking survey design into account.

**Results:**

Twelve months prior to being surveyed, the prevalence of suicidal behaviors was 9.3%, 6.5% and 5.9% for suicidal ideation, suicidal plan and suicidal attempt, respectively. Females were overrepresented in attempts (61.2%).

Several self-reported characteristics such as suicide ideation (69%), anxiety (28%), and loneliness (30%) were significantly different between the attempters of suicide and non-attempters (p less than 0.05). Also, some suicide-related behaviors such as having planned a suicide (52%), being bullied (21%), involved in a physical fight (29%), serious injury (29%), early sexual debut (8.5%), alcohol use at early age (21%), alcohol use in the past 30-days (12%), and being physically attacked (30%) differed by suicide category (p less than 0.05). Compared to those who did not report attempting suicide, attempters were more likely to have suicide ideation (OR=10.58; 95% CI 5.10, 21.97); have planned suicide (OR=9.82; 95% CI 4.60, 20.96); or sustained serious injury (OR=4.01; 95% CI 2.03, 7.93) within the recall period.

**Conclusions:**

This study provided evidence, which overall confirm that the psycho-social environment in school settings modify suicidal behavior. The results, taken together emphasize the importance of the school environment on the development of school attending adolescents. Where possible, the results may provide additional information on which self-reported behaviors represent avenues for potential preventive programming.

## Introduction

Suicide is the act of intentionally ending one's own life.^1^ It is a global phenomenon that is not confined to a particular gender, age, ethnicity, nationality or religion. The World Health Organization (WHO) reports that about 800,000 people commit suicide every year; which translates to one suicide every 40 seconds.^2^ A total of 29% (569 cases out of 1959) of mortality having an external cause occurred as a result of suicide between 2007-2011.^2,3^ Suicide ranks the second most important cause of mortality during the second and third decades of life.^4^ In particular, it represents the third top cause of death among 15-19-year-olds for both sexes.^5^ Furthermore, for each adult who commits suicide, there are other 20 people who attempt.^6^

Adolescence, a phase of major development, involves a profound extent of change in all areas of development—biological, cognitive, psychosocial, emotional, and relational.^7^ Therefore, the adolescence becomes of vital importance for its critical development and onset of suicidal behaviors.^4,8,9^ Suicidal thoughts and behaviors (hereafter referred to as “suicidal behaviors”) are categorized into three classes: suicide ideation (SI) (thoughts of engaging in behavior intended to end one's life); suicide plan (SP) (formulation of a specific method through which one intends to die; and suicide attempt (SA) (engagement in potentially self-injurious behavior in which there is at least some intent to die).^1^ Among countries with representative data on suicide, the prevalence ranges from 2.3% in Vietnam to 28.3% in Benin.^10-22^

Co-occurrence of several distal risk factors (such as socioeconomic factors, psychiatric diagnosis, previous suicide attempts, family history of suicide/attempts and so on) and proximal risk factors (recent onset of suicidal thoughts, hopelessness, existence of a suicide plan, access to firearms, a major loss, stressful event, imprisonment and so forth) is demonstrated to be deeply associated with suicidal behaviors.^17,23-25^ As a solution to having approximately 14% of the British people report feeling lonely, the UK established a Ministry for Loneliness - the first of its kind in the world.^23^ Although the majority of suicides (76% in 2012, 79% in 2019) are committed by residents in low- and middle-income countries (LMICs) with 84 percent of the population,^5,26^ high income countries (HICs) including Brunei Darussalam (hereinafter is referred to as Brunei)—located in the WHO Western Pacific Region, also suffers from its side effects.^27,28^ Furthermore, the majority of the problem has moved to Asia (accounts for higher rate of suicide in the world) from Western Europe.^29^ With approximately one-fourth of the world’s population located in the 11 countries of the WHO South East Asia Region, almost 40% of the suicide happens in the region.^29^ With the prevention of suicidal behavior being a longstanding global public objective,^5^ prevention policy should be based on local evidence. Therefore, with regard to the importance of suicide, and the dearth of specific research in the region among adolescents, the aim of this study was to determine the prevalence of suicidal behaviors and associated factors among Bruneian adolescents.

## Methods

**Setting**

The data for this study were collected in the Nation of Brunei (Brunei Darussalam), a HIC located in South East Asia, bordering Malaysia and the South China Sea. Based on mid 2018 population estimates, Brunei has a population of 442,400 people (233,400 male (52.8%)).^27^ According to the age distribution, almost 20.7% and 17.9% of the population were under age 15 years.^27^ Life expectancy in 2018 was 75.3 for males and 77.6 for females.^30^

This work represents a secondary analysis of the Brunei contribution to the 2014 Global school-based student health survey (GSHS). It was developed by the WHO and the United States Centers for Disease Control and Prevention (CDC).^10^ The tool’s internal consistency (Cronbach’s alpha) were differed but acceptable in different countries, with 0.69,^31^ 0.642 (relational dimensions), 0.737 (structural dimensions), 0.843 (pedagogical dimensions).^32^ The target population consisted of school-attending adolescents aged 13-17 years. A two-stage cluster sampling procedure yielded data which was representative of all school-attending adolescents in Brunei. The first stage was defined as probable selection of the schools which was proportional to enrollment size. At the next stage, randomly selected classes were sampled, of which all students were eligible to contribute. In Brunei, with 2599 respondents, the response rates were 100% and 65% at school and student levels, respectively. 

In accordance with the GSHS study protocol, questionnaires were administered to all eligible volunteers in an anonymous manner. Written permission had been attained from all engaged schools and all sampled classrooms’ teachers. Since Bruneian adolescents are deemed competent to give informed consent at the age of 18,^33^ an assent was also received from their parents.^10^

**Measurements**

The dependent variable ‘SA’ was derived from this question: “During the past 12 months how many times did you actually attempt suicide?” Response options ranged from “0 time”, “1 time”, “2 or 3 times”, “4 or 5 times”, and “6 or more times”. These responses were dichotomized into ‘zero’ corresponding to “0” attempt (N = 2,429), and the rest were lumped together as ‘1’ (N = 153), representing those students who had attempted suicide once or more during the preceding 12 months. This information was missing for 17 records. For 8 records information on gender was missing, and for 3 records information on age was missing (with one record missing information on both). The analysis presented in this paper did not exclude any cases. The current study used the term of recall period as an easily remembered point in time that refers to several periods like 7 days, 30 days, past year and “within the school year” in a variety of questions.^34^ We investigated fifteen variables at the individual level and four variables at the social level. Details on how these variables were created are provided in Table 1.

**Table 1:** Variables studied in GSHS in Brunei, 2014.

Survey question	Coding	Variable
**Demographic variables**		
How old are you?	11 or less to 18 years or above (coded continuous)	Age
What is your sex?	Male (1)	Sex
	Female (0)	
**Dependent variable**		
During the past 12 months, how many times did you actually attempt suicide?	0 times (no)	Suicide Attempt
	1 to 6 or more times (yes)	
**Independent variables**		
During the past 12 months, did you ever seriously consider attempting suicide?	Yes (1)	Suicide Ideation
	No (0)	
During the past 12 months, did you make a plan about how many times you would attempt suicide?	Yes (1)	Suicide planning
	No (0)	
During the past 12 months, how often have you been so worried about something that you could not sleep at night?	Most of the time/always (yes)	Anxiety
	Never/rarely/sometimes (no)	
During the past 12 months, how often have you felt lonely	Most of the time/always (yes)	Loneliness
	Never/rarely/sometimes (no)	
During the past 30 days, how often did you go hungry because there was not enough food in your home?	Most of the time/always (yes)	Food deprivation
	Never/rarely/sometimes (no)	
How many close friends do you have?	0, 1, 2, and 3 or more friends (coded continuous)	Close friends
During the past 30 days, on how many days were you bullied?	3 or more days (bullied)	Bullying victimization
During the past 30 days, how were you bullied most often?	I was hit, kicked, pushed, shoved around, or locked indoors	Physical bullying
Which of your parents or guardians use any form of tobacco?	Father, mother, or male/female guardians (yes) Neither/I don't know (no)	Parental tobacco use
During the past 7 days, on how many days have people smoked	0 days (0)	Days people smoked in presence during the last week
	1–2 days (1)	
	3–4 days I(2)	
	5–7 days (3) (coded continuous)	
During the past 12 months, how many times were you in a physical fight?	0 – 1 times (no)	Physical fight
	2 – 12 or more times (yes)	
During the past 12 months, how many times were you seriously injured?	0 – 1 times (no)	Serious injury
	2 – 12 or more times (yes)	
How old were you when you had sexual intercourse for the first time?	Never had sexual intercourse or had sexual intercourse at age 15 or above (no)	Early sexual debut
	Had sex before age 15 (yes)	
During your life, with how many people have you had sexual intercourse?	6 people or more (coded continuous)	Lifetime sexual partners
How old were you when you had your first drink of alcohol other than a few sips?	Never had an alcoholic drink or had it at age 16 and above (no use of alcohol)	Alcohol use at early age
	Had a drink age 15 or below (use of alcohol)	
During the past 30 days, on how many days did you have at least one drink containing alcohol?	0 days (no)	Alcohol use in the past 30 days
	1 – 30 days (yes)	
During the past 12 months, how many times were you physically attacked?	0 – 1 times (no)	Physically attacked
	2 – 12 or more times (yes)	

**Statistical Analysis**

Univariate analyses characterized the distribution of each selected variable among those who never attempted suicide compared to those who attempted one or more times. This was followed by bivariate analyses examining associations among school-attending adolescents students who never attempted suicide versus those who reported attempting suicide one or more times and the selected independent variables. The independent samples t-test was used for the variables age, number of friends, number of days others smoked around the respondent, and number of lifetime sex partners. For the additional variables, the Chi-Square test was used. All statistically significant independent variables were included in the multivariate analysis for the dependent variable ‘SA’ using multiple logistic regression model. P-values of less than 0.05 were regarded as being statistically significant.

The logistic regression model included all independent variables found to be statistically significant at p<0.05 in the bivariate analyses. Odds Ratios (OR), including their 95% and 99% confidence intervals, are reported for the strength and direction of associations between being involved in physical fights and the other studied factors; Stata version 15 (StataCorp LP, College Station, TX, USA, 2017) was used for the data analysis. All proportions - expressed in percentages – are weighted. 

## Results

The current study investigated several variables in which were potentially related with suicidal behavior among school-attending adolescents in Brunei. Of 2599 participants, 49.9% were female. The mean age of the participants was 14.7 years old (95% CI: 14.5-14.8).

Within the recall period, 5.9% (unweighted count: 153) of participants reported having attempted suicide in the past twelve months (95% CI: 4.95-7.01), most of whom were female (61.2%). During the 12 months preceding the survey, 9.3% reported having seriously considered attempting suicide (95% CI: 7.93-10.90). While 6.5% reported having made a plan about how to attempt suicide (95% CI: 5.35-7.92). Within the same period of recall, 10.4% reported been so worried about something, either most of time or always, so that they could not sleep at night. Almost 12% of respondents felt lonely either most of the time or always in the past twelve months. In the 30 days preceding the survey, 6.7% of participants had gone hungry always or most of time because there was not enough food in home. The mean number of friends reported was 2.76 (SD: 0.67). 

Within the 30 days prior to being surveyed, 9.0% and 1.4% of respondents reported being bullied during three or more days and bullied physically, respectively. During the seven days prior to being asked, on average, on 0.91 days (SD: 1.4) others had smoked in the presence of respondents. During the past 12 months, 13.9% respondents reported being involved in two or more physical fights, and 13.5% reported having sustained serious injuries on at least two occasions. Early sexual debut, defined as having had sex before the age of 15, was reported by 2.9% of respondents. Regarding the number of lifetime sexual partners, of those who were sexually active (n=92 out of 2559), 31 out of 92 respondents reported having had one sexual partner while another n=31 reported having more than 2. Alcohol use at an early age, defined as having an alcoholic drink before the age of 16, was reported by 9.0%,while 4.5% reported having had at least one drink containing alcohol in the 30 days prior to being asked for one or more days. Finally, 18.5% of respondents stated that they had been physically attacked two or more times within a 12 month period of recall.

Table 2 shows the weighted distribution of selected factors according to the SA category. The bivariate analyses indicated statistically differences between participants who had attempted suicide and those who had not, within all but one of the selected variables i.e. parental tobacco use.

**Table 2:** Comparison of factors by suicide attempt in GSHS, Brunei, 2014.

Variable	No suicide attempt (n = 2,429)	One or more suicide attempts (n = 153)	P-value
Age (SD)	14.63 (1.37)	14.97 (1.49)	0.013
Sex (male)	50.9	38.8	0.012
Suicide ideation	5.7	69.4	<0.001
Suicide planning	3.7	52.8	<0.001
Anxiety	9.3	28.3	<0.001
Loneliness	11.7	30.4	<0.001
Food deprivation	6.3	12.8	0.003
Close friends (SD)	2.77 (0.66)	2.59 (0.82)	0.037
Bullying victimization	8.3	21.2	<0.001
Physical bullying	1.3	3.8	0.031
Parental tobacco use	31.7	35.8	0.240
Days people smoked in presence during the last week	0.88 (1.34)	1.37 (1.60)	0.002
Physical fight	13.0	29.2	<0.001
Serious injury	12.7	28.8	<0.001
Early sexual debut	2.5	8.5	<0.001
Lifetime sexual partners	0.80 (0.55)	0.35 (1.08)	0.006
Alcohol use at early age	8.2	20.8	<0.001
Alcohol use in the past 30 days	4.0	12.4	<0.001
Physically attacked	17.7	30.0	<0.001

All variables are expressed as proportions (in %) with the exception of age, close friends, days people smoked in presence during the last week, and life time sexual partners (mean and standard deviation).

Table 3 provides the results of the adjusted analysis i.e. after adjusting for all the covariates that were found to be statistically significant in the bivariate analysis. Out of the eighteen explanatory variables used, three were found to be statistically significant at p<0.05 as well as p<0.01. Compared to those who did not report having attempted suicide, those who had attempted suicide were more likely to have had SI (OR 10.58; 95% CI 5.10, 21.97); planned suicide (OR 9.82; 95% CI 4.60, 20.96); and had sustain serious injury (OR 4.01; 95% CI 2.03, 7.93). The goodness-of-fit test revealed that this was a good multivariate logistic model for suicide attempts in Bruneian students (F: 0.96; p-value: 0.4798). 

**Table 3:** Multivariate analysis of variables associated with suicide attempts in GSHS, Brunei, 2014.

Variable	Adjusted Odds Ratio	95% CI (99% CI)*	P-value
Sex	0.70	0.41, 1.22	0.209
Age	0.97	0.82, 1.15	0.728
Suicide ideation	10.58	5.10, 21.97 (4.02, 27.81)	<0.001
Suicide planning	9.82	4.60, 20.96 (3.60, 26.78)	<0.001
Anxiety	1.22	0.63, 2.36	0.550
Loneliness	1.24	0.67, 2.32	0.490
Food deprivation	1.40	0.51, 3.85	0.507
Close Friends	0.93	0.60, 1.45	0.759
Bullying victimization	0.83	0.36, 1.92	0.657
Physical bullying	0.91	0.17, 4.77	0.908
Days people smoked in presence during the last week	1.14	0.96, 1.35	0.130
Physical fight	1.38	0.78, 2.44	0.259
Serious injury	4.01	2.03, 7.93 (1.63, 9.88)	<0.001
Early sexual debut	0.44	0.08, 2.29	0.326
Lifetime sexual partners	1.16	0.69, 1.96	0.577
Alcohol use at early age	1.19	0.38, 3.71	0.759
Alcohol use in the past 30 days	1.06	0.29, 3.93	0.926
Physically attacked	0.62	0.36, 1.07	0.083

Only those factors found statistically significant in bivariate analysis were used in this model. * 95% and 99% CI indicate 95% and 99% Confidence Intervals.

## Discussion

This study provided estimates on the prevalence of and correlates for suicidal behaviors and selected self-reported measures of psychosocial health among school-attending adolescents aged 13-17 in Brunei Darussalam. 

**Correlates for suicide attempts**

In the present study, suicide attempters demonstrated significantly higher odds of having a serious injury, SI and SP in accordance with previous research. Self-reported SI was ten times more likely among attempters than non-attempters.^35^ Suicide planning was about ten times more likely among adolescent attempters than non-attempters. A study carried out in Malawi reported a somewhat lower association of four times more among attempters.^10,35^ Having had a serious injury was about four times more likely among adolescents attempters than non-attempters, similar to a study conducted in Malawi.^10^

Our findings did not demonstrate a significant relationship between attempt and being physically bullied, having anxiety and more lifetime sexual partners, which was a key finding in previous research.^10,13,36^ The pattern of suicidal behavior differs from the pattern in LMICs, in spite of the same research methodology.^36^

**Suicidal behaviors**

The current study examined suicidal behaviors comprising SA, SI and SP^37^ with the following description.

**Attempting suicide**

The prevalence of SA among Bruneian school-attending adolescents (5.9%) was low compared to the rate in low and middle income countries such as Nepal (10.33%), Kuwait (18.1%), and some countries in African WHO Region and in South East Asia Region (crude suicide rate: 12.9%) during the recall period.^4,10-14^ It is worth noting that Brunei is one of 25 countries with specific laws and punishments for attempted suicide.^38^ Prohibiting regulations can play major role in inhibiting people from doing illicit behaviors. A society’s socioeconomic condition appears to indicate a negative association with intentional injuries, since fatal injuries in rural areas were 1.5 times higher compared to urban areas, even with respect to segregated intentional and unintentional mortalities, rural areas maintained higher incidence crude mortality rate.^3^ Moreover, the frequency of SA is strongly related to suicide.^4^ On the other side, Brunei presented higher percentage for SA when comparing with estimate of Indonesia as a Middle-Income Country, with 2.46% based on a sample of 8634 students in 2015,^15^ and of a prospective study of 7,072 adolescents 1.6% in China as an Asian upper middle income country.^16^

Adolescents in Brunei also had a lower rate of suicide (4.75 per 100,000, based on data in 2012) compared to other HICs,^39^ while according to data from the WHO, it had a comparable prevalence to (6 per 100,000 population in 2012).^40^ Among 15- to 24-year-olds, suicide is the third leading cause of death in the United States. The population-based rate of non-firearm related suicide per 100,000 was reported for other HICs such as the U.S. (6.1), Sweden (11), Norway (9.5), Australia (10.2), Finland (14.5), and Japan (23.1).^41^ Although Japan is an HIC, and less populous compared with the U.S., it has a higher suicide rate. 

Female respondents (61.2%) attempted suicides more than male ones, similar to some countries in African Region as well as South East Asia Region.^4,10,11,42^ Female university students reported lower rate of SA than male students in Spain.^17^ However, some studies revealed male overrepresentation in suicide rates and female overrepresentation in SAs.^4,43,44^ Some studies indicated reasons for the lower rate of female suicide regarding the suicide method. Overdoses attempted mostly by women are treated more effectively than others like hanging, which is used in attempts more often by males.^45^ The present study did not examine the method for suicide attempt. 

**Suicidal ideation and plan**

In Brunei, the one-year prevalence of suicidal ideation among the sampled population (9.3%) was similar to the rate in Spain (9.9%), lower than the rate in LMICs such as countries in African region, South-East Asian Region, and Western Pacific Region.^10-13,17,18,46^ The rate of suicidal ideation was approximately similar to the rate in the GSHS of Thailand (8.8%) and China (8.1%) but higher than the rate in countries in western pacific region, Iran (4%), and Indonesia (4.75%).^15,16,19-22^

Compared with suicide attempt, suicidal ideation is reported more prevalent,^1^ even in Bruneian lower frequencies. Adolescents with suicidal ideation are approximately 12 times more likely to attempt suicide by the age of 30 years,^9^ thus the development of prevention strategies which take into account suicide as encompassing a spectrum of risk behaviors and actions is of significant importance. Additionally, in the present sample, about 6% of respondents reported having made a plan about how to attempt suicide which is comparable to the statistics from Spain (5.6%) with a majority of the respondents being female^17^ and higher than estimates from a Chinese study (2.1%).^16^ It is much lower rate than the estimation by the 2003 Youth Risk Behavior Surveillance (YRBS) survey (16.9% SI and 16.5% SP in 32 states) in the US may imply differences in the study design.^1,45^

**Psychosocial factors**

In this study, we examined some psychosocial factors including worry about something, self-reported anxiety, loneliness, being bullied, being involved in physical fight and alcohol use in relation to the respondents’ suicidal behaviors. The 12-month prevalence of self-reported anxiety was 10.4% of Bruneian school attending adolescents. More anxiety was felt by adolescents attempted suicide, similar to some studies.^11,13,45^ Studies indicated a significant association between a psychiatric disorder and SAs.^4,47^ Neuropsychiatric disorders contribute to about considerable percentage of the global burden of disease.^48^ Having felt lonely, either most of time or always in the twelve months preceding the survey was reported by 12.7% of respondents. Those who reported loneliness were more likely to report suicidal behaviors, similar to a previous study finding in Benin and Tanzania.^11,13,49^ According to the interpersonal theory of suicide, feeling lonely (thwarted belongingness), and a sense that one is a burden on others (feeling perceived burdensomeness) are factors which may increase the potential for suicidal thoughts.^50^

In line with this finding, depression and severe stress were demonstrated by other studies as also being linked with suicidal thoughts.^35,47^ Having clinical disorders like depression is known as a reason for transition to attempt from ideation in their youth.^9^ Bruneian adolescents (13.9%) reported being less involved in a fight than Benin adolescents (48.3%).^11^ It may be indicative of higher gang-related violence among adolescents in Benin. About large percentage of respondents (18.5%) reported been physically attacked, two or more times, in the past 12 months. Adolescents who attempted suicide had reported being physically attacked more often than those who did not, similar to some studies.^4,11^ Being victimized and physically attacked is a risk factor for suicidality.^11,45^

Suicide attempters in this study reported more alcohol use at early age and in the past 30 days than non-attempters (p-value<0.001).^51^ The alcohol misuse in Bruneian adolescents is much lower than in Benin.^11^ It may be due to the prohibition of alcohol sale, especially to individuals at risk could have an important effect in reduction of thinking about suicide.^28^ Furthermore, according to the model of stress-diathesis, risk factors like alcohol use imposes bigger stress and lead person to commit suicide so as to escape from problems,^11^ of most common risk factors, alcohol use/substance abuse represent high percentage in suicide as mentioned by studies.^13,35,45,47^

**Strengths and limitations**

Several aspects of this study have contributed to the reliability of the findings. It includes a large number of school-attending adolescents' representative for the country as a whole. The questionnaire used in all of the GSHS data collection efforts had been developed and tested internationally. However, the findings presented here must be viewed in light of several limitations. The data are cross-sectional which limits the ability to examine causality. In spite of total sampling of in-school adolescents, those who did not attend school, or who were absent on the day of the survey are not represented in these data. Also, it is also estimated that the results may be underestimations of the phenomena under study due to the sensitive nature of some of the questions, although all participants were informed of anonymous nature of the responses. The social and cultural taboos around the subject of suicide in particular, may have had an impact on the responses.^26^

## Conclusion

The current study provided evidence confirming that the psycho-social environment in school settings modifies suicidal behavior among school-attending adolescents. The results, taken together underline the importance of the school environment on adolescents’ development. Where possible, the results may provide additional information on which self-reported behaviors represent avenues for potential preventive programming. Upcoming research with experimental or qualified observational methodologies are suggested too.

**Acknowledgments**

This paper uses data from the Global School-Based Student Health Survey (GSHS). GSHS is supported by the World Health Organization and the US Centers for Disease Control and Prevention. The authors thank the Republic of Brunei Ministry of Health; the World Health Organization (Geneva, Switzerland); and the Centers for Disease Control and Prevention (Atlanta, GA, USA). We would also like to thank all participants, the survey officers and students. Author M.L.W. was supported by the Alexander von Humboldt-Stiftung, Bonn, Germany.
